# Supplementary material for: In vitro efficacy of Artemisia extracts against SARS-CoV-2
Source: Virol J. 2021 Sep 8;18:182. doi: 10.1186/s12985-021-01651-8 (PMC8424155; doi:10.1186/s12985-021-01651-8)
Supplement: Supplementary file 1 — Additional file 1. The following are available: Figure S1: Viability of CRFK cells being cultured in presence of the samples for 45 min. Values are expressed as mean ±SD, n=3. Figure S2: Inhibitory activity of Covid-Organics against two different coronaviruses. Values are expressed as mean ±SD, n=3. Figure S3. Viability of VeroE6 cells being cultured in presence of the samples for 24 h. Values are expressed as mean ±SD, n=3. Table S1: List of dried leaves and compounds from various places and their inhibitory activity against FCoV. [file 12985_2021_1651_MOESM1_ESM.docx]

*In vitro* efficacy Artemisia extracts against SARS-CoV-2

Chuanxiong Nie^1,2^, Jakob Trimpert^1^, Sooyeon Moon^3^, Rainer Haag^2^, Kerry Gilmore^3,4^, Benedikt B. Kaufer^1^*, and Peter H. Seeberger ^2,3^*

1 Freie Universität Berlin, Institute for Virology, Robert von Ostertag-Str. 7-13, 14163 Berlin, Germany chuanxnie@zedat.fu-berlin.de, Jakob.Trimpert@fu-berlin.de; Benedikt.Kaufer@fu-berlin.de

2 Freie Universität Berlin, Department of Chemistry and Biochemistry, Takustrasse 3, 14195 Berlin, Germany haag@zedat.fu-berlin.de

3 Max-Planck Institute for Colloids and Interfaces, Department for Biomolecular Systems; Am Mühlenberg 1, 14476 Potsdam, Germany Soo-Yeon.Moon@mpikg.mpg.de, kerry.m.gilmore@uconn.edu, Pe-ter.Seeberger@mpikg.mpg.de

4 Current address: Department of Chemistry, University of Connecticut, 55 N. Eagleville rd. Storrs, CT, USA 06268.

* Correspondence: peter.seeberger@mpikg.mpg.de; Tel.: +49-331-567-9300 (P.H.S.); Benedikt.Kaufer@fu-berlin.de; Tel.: ++49-30-838-51936 (B.K.);

.

**SUPPLEMENTARY INFORMATION**

Contents

[1. Reagents and Materials 2](#_Toc63782017)

[2. Extraction of *Aremisia* leaves 3](#_Toc63782018)

[General procedure: Extraction using distilled water 3](#_Toc63782019)

[3. References 4](#_Toc63782020)

# 1. Reagents and Materials

Solvents were obtained from commercial suppliers and used without further purification. Information of dried leaves were listed below (Table S1). Samples are packed and stored under ambient conditions. For further details, please contact companies listed in the Table S1. Artemisinin was previously prepared and purified by crystallization using published protocols.^S1^ Crystals were ground prior to use. Covid-Organics was used without further purification. Filter paper used was Rotilabo type 113A, diameter 240 mm, obtained from Carl Roth.

| **Sample** | **Year** | **EC50 _FCoV_** | **CC50 _CRFK_**  **(mg/mL)** | **Selectivity index (FCoV)** | **Affiliation** |
| --- | --- | --- | --- | --- | --- |
|  |  | **(mg/mL)** |  |  |  |
| A. annua (Germany) | 2019 | 0.78 ± 0.20 | 8.52 ± 3.72 | 10.92 | Teemana |
| A. annua (Nigeria) | 2019 | 1.13 ± 0.31 | 9.40 ± 4.92 | 8.32 | Lucile Cornet Vernet |
| A. annua (Chad) | 2019 | 1.03 ± 0.30 | 8.81 ± 11.15 | 9.08 | Lucile Cornet Vernet |
| A. annua (Madagascar) | 2019 | 0.97 ± 0.27 | 11.15 ± 8.07 | 11.49 | Lucile Cornet Vernet |
| A. tridentata (USA, Utah) | 2019 | 0.46 ± 0.12 | 7.21 ± 2.60 | 15.67 | Lucile Cornet Vernet |
| A. absinthium (France) | 2019 | 0.83 ± 0.23 | 10.42 ± 5.03 | 12.55 | Lucile Cornet Vernet |
| A. annua (Togo) | 2019 | 0.69 ± 0.15 | 13.37 ± 10.55 | 19.37 | Lucile Cornet Vernet |
| A. annua (Congo RDC) | 2020 | 0.40 ± 0.12 | 26.17 ± 34.82 | 65.42 | Lucile Cornet Vernet |
| A. annua (France) | 2019 | 0.75 ± 0.17 | 7.08 ± 6.28 | 9.44 | Lucile Cornet Vernet |
| A. annua (Burkina Faso) | 2019 | 0.44 ± 0.09 | 5.64 ± 3.58 | 12.82 | Lucile Cornet Vernet |
| A. afra (Chad) | 2019 | 0.44 ± 0.07 | 7.55 ± 4.38 | 17.16 | Lucile Cornet Vernet |
| A. afra (Benin) | 2019 | 0.33 ± 0.07 | 8.74 ± 4.28 | 26.48 | Lucile Cornet Vernet |
| A. afra (France) | 2019 | 0.55 ± 0.17 | 9.22 ± 3.58 | 16.76 | Lucile Cornet Vernet |
| A. afra (France) | 2015 | 0.63 ± 0.10 | 20.44 ± 15.62 | 32.44 | Lucile Cornet Vernet |
| A. annua var. (Brazil) | 2020 | 1.76 ± 0.42 | 10.52 ± 5.64 | 5.98 | University of Campinas |
| Artemisinin | - | 6.69 ± 1.05 | 15.93 ± 8.22 | 2.38 | - |
| A. annua var. CPQBA 1  alcoholic extract (Brazil) | 2020 | 0.002 ± 0.001 | 0.94 ± 0.32 | 470.00 | University of Campinas |
| A. Annua (USA, Kentucky) | 2019 | 1.76 ± 0.54 | 3.57 ± 1.10 | 2.02 | ArtemiLife Inc. |
| Covid-Organics (Madagascar) | 2020 | 76.81 ± 11.60  (% to raw drink) | 41.13 ± 12.27  (% to raw drink) | 0.54 | Malagasy Institute for Applied Research |

**Table S1.** List of dried leaves and compounds from various places and their inhibitory activity against FCoV.

# 2. Extraction of *Aremisia* leaves

## General procedure: Extraction using distilled water

Distilled water (10 mL, VWR) were added to an Erlenmeyer flask (50 mL). The solvent was heated to 90 ˚C using the hot plate. Dried leaf material (1 g) was added to the temperature-stable water and allowed to keep for two minutes at 90 ˚C and then 20 minutes at room temperature. The samples were filtered using filter paper and the solid material was washed with room temperature water (20 X 2 mL). The filtered solution was then dried using a rotary evaporator at least 2 hours. Dried samples were dissolved in DMSO (3 mL). Sonication needed for maximal solvation. The sample was filtered through a syringe filter (Chromafil® xtra RC0.45). The sample was stored at -10 °C until use.

For the alcohol extraction sample, tincture mother was prepared with the same material CPQBA 1, into a rate of 1:10, it means: 100g of dried leaves of A. annua var. CPQBA 1 to 1000mL of ethanol 70° shaking it once a day by 20 days. 2mL of alcoholic extract was dried by rotary evaporation dissolved in DMSO (3 mL). The sample was stored at -10 °C until use.

**Artemisinin.** Artemisinin (500 mg) was dissolved in DMSO (3.0 mL). The solution was transferred with a 1000 µL pipette to a 1.5 mL Eppendorf snap-close vial. Concentration of example sample: 167 mg/mL. The sample was stored at -10 °C until use.

**Covid-Organics.** Covid-Organics (50 mL) was dried by rotary evaporation dissolved in DMSO (3 mL). The sample was stored at -10 °C until use.


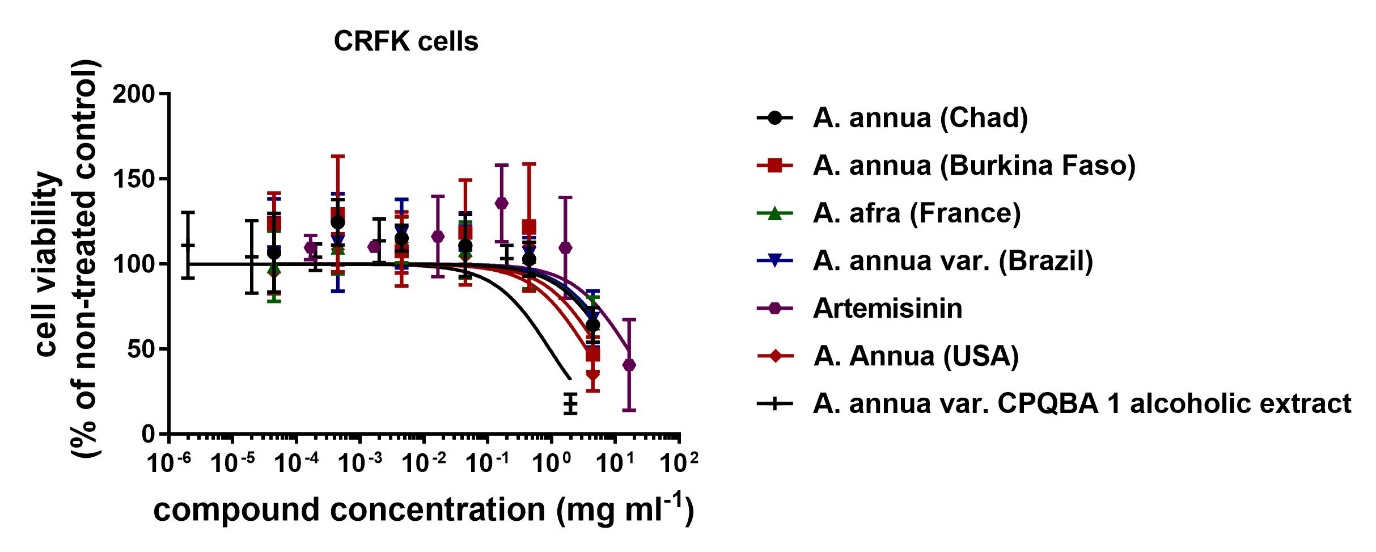


Figure S1. Viability of CRFK cells being cultured in presence of the samples for 45 min. Values are expressed as mean ±SD, n=3.


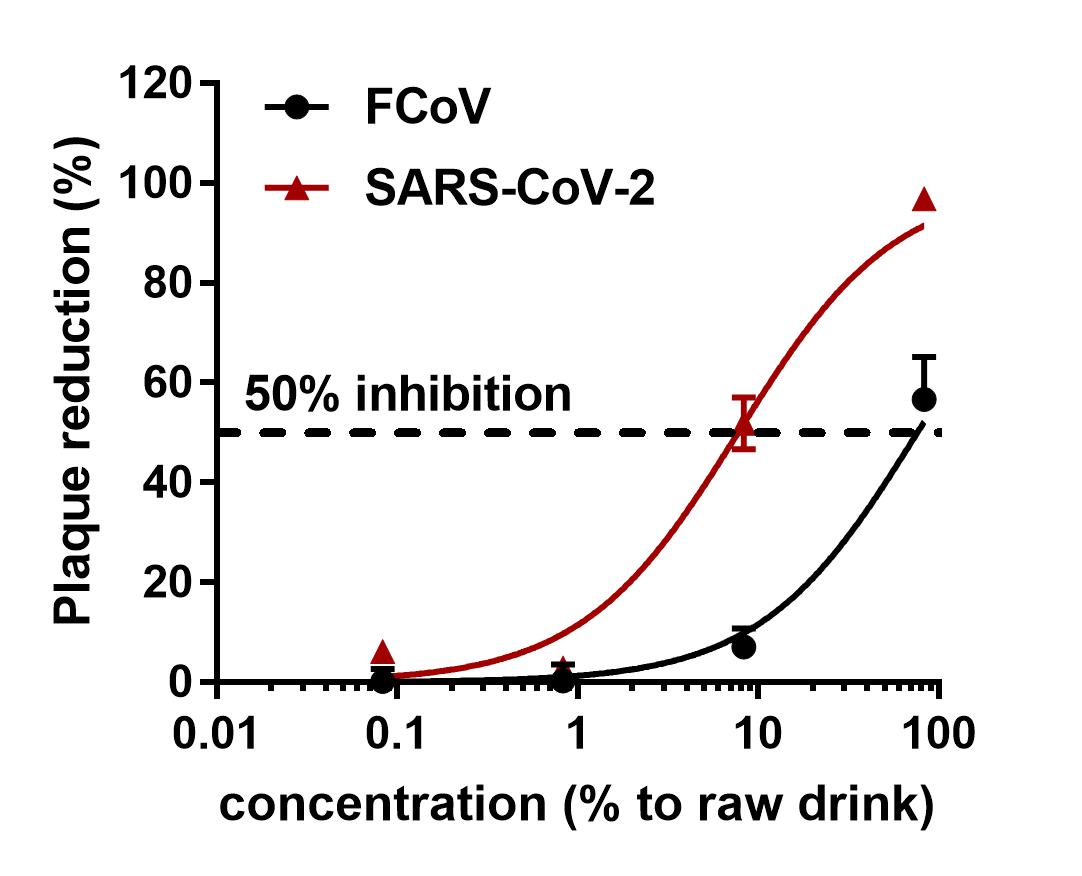


Figure S2. Inhibitory activity of Covid-Organics against two different coronaviruses. Values are expressed as mean ±SD, n=3.


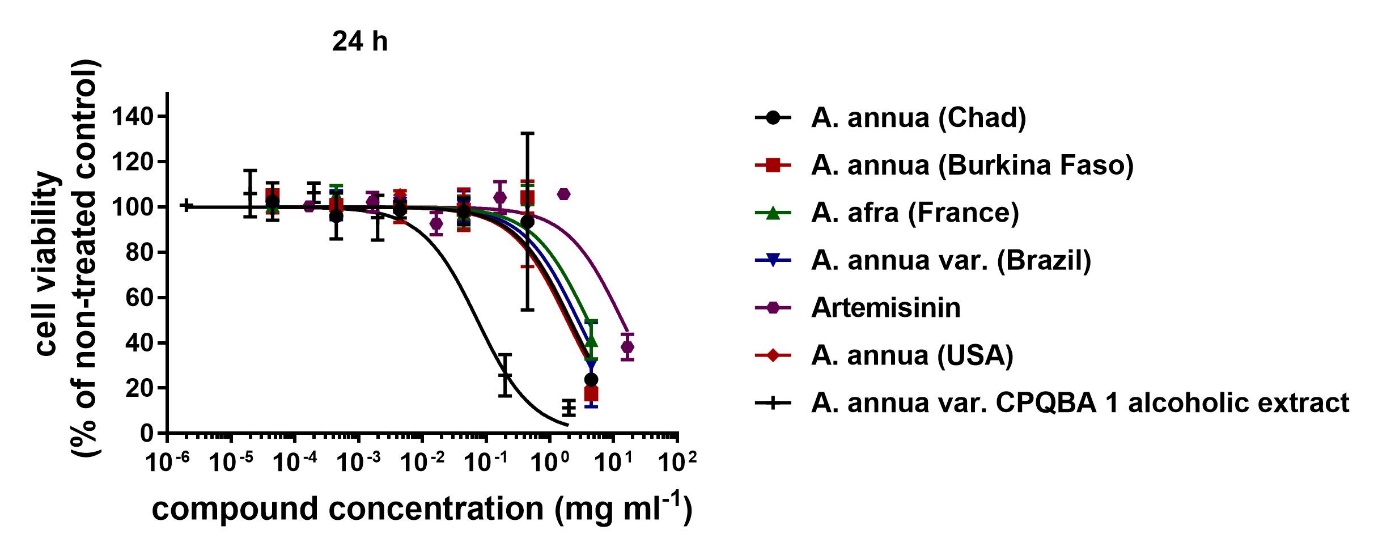


Figure S3. Viability of VeroE6 cells being cultured in presence of the samples for 24h. Values are expressed as mean ±SD, n=4.

# 3. References

S1) Horváth, Z.; Horosanskaia, E.; Lee, J. W.; Lorenz, H.; Gilmore, K.; Seeberger, P. H.; Seidel-Morgenstern, A. Recovery of Artemisinin from a Complex Reaction Mixture Using Continuous Chromatography and Crystallization. *Org. Process Res. Dev.* **2015**, *19*, 624-634.

S2) Bussmann, B. M.; Reiche, S.; Jacob, L. H.; Braun, J. M.; Jassoy, C. Antigenic and cellular localization analysis of the severe acute respiratory syndrome coronavirus nucleocapsid protein using monoclonal antibodies. *Virus Res.* **2006**, *122*, 119-126. DOI: 10.1016/j.virusres.2006.07.005.

S3) von Einem, J.; Schumacher, D.; O’Callaghan, D. J.; Osterrieder, N. The α-TIF (VP16) homologue (ETIF) of equine herpesvirus 1 is essential for secondary envelopment and virus egress. *J. Virol.* **2006**, 2609-2620. DOI: 10.1128/JVI.80.6.2609–2620.2006.
